# Deficiency of Yes-Associated Protein Induces Cataract in Mice

**DOI:** 10.14336/AD.2018.0910

**Published:** 2019-04-01

**Authors:** Qing He, Yuhao Gao, Tongxing Wang, Lujun Zhou, Wenxia Zhou, Zengqiang Yuan

**Affiliations:** ^1^State Key Laboratory of Brain and Cognitive Sciences, Institute of Biophysics, Chinese Academy of Sciences, Beijing 100101, China; ^2^The Brain Science Center, Beijing Institute of Basic Medical Sciences, Beijing 100850, China; ^3^College of Life Sciences, University of Chinese Academy of Sciences, Beijing 100049, China; ^4^Beijing Institute of Pharmacology and Toxicology, Beijing 100850, China; ^5^State Key Laboratory of Toxicology and Medical Countermeasures, Beijing 100850, China

**Keywords:** cataract, Yap, lens epithelial cells, cell proliferation, cell senescence

## Abstract

Cataract is a major cause of blindness worldwide, its complicated and unclear etiopathogenesis limit effective therapy. Here, we found that Yap, a downstream effector of the Hippo pathway, is specifically expressed in lens epithelial cells and Yap conditional knockout (cKO) in the lens leads to cataract. Histologically, Yap deficient lens show fewer epithelial cells, retention of nuclei and accumulation of morgagnian globules in the transitional zone and the posterior area. Mechanistically, GFAP-mediated Yap cKO leads to the reduced proliferation of epithelial cells, delayed fiber cell denucleation and increased cellular senescence in lens. Further RNA profiling analysis reveals Yap cKO results in a significant alteration in gene transcription that is involved in eye development, lens structure, inflammation, cellular proliferation and polarity. Collectively, our data reveal a novel function of Yap in the lens and links Yap deficiency with the development of cataract, making Yap a promising target for cataract therapy.

Cataract is the most common disease that causes blindness with a clouding lens [1]. The development of cataract is the combination result of multiple factors, and the exact etiopathogenesis is unclear. So far, the formation of cataract can be attributed to a combination of factors such as dysregulated proliferation [2] and apoptosis [3] of lens epithelial cells, abnormal lens fiber cell differentiation and denucleation [4-6], cellular senescence [7], oxidative stress [8], and upregulated inflammatory factors in the eyes [9].

Yap, the core downstream transcription co-activator of Hippo signaling pathway, plays a key role in tissue homeostasis and organ size control by regulating proliferation, differentiation and apoptosis of cells [10-12]. Recent work has indicated that Yap controls DNA replication timing and genomic stability of retinal stem cells [13]. Specifically, Yap is essential for cell cycle progression in retinal progenitors and fate acquisition in retinal pigment epithelium (RPE) in developing murine eyes [14, 15]. Yap has also been reported to be vital to the proliferation and differentiation of lens progenitor cells [16], and fibroblast growth factor (FGF) could regulate nuclear Yap expression in the cells [17]. Recently, the role of Yap in the development of eye pathology has been reported. However, the function of Yap-mediated signaling in lens epithelial cells and the development of cataract remains elusive.

Here, we first characterized the expression of Yap and GFAP-Cre recombinase in the developing and adult mouse lens and found that both Yap and GFAP are mainly expressed in the lens epithelium. Next, we demonstrated that conditional knockout (cKO) of Yap using GFAP-Cre leads to the development of cataract, with 69.4% incidence at 1.5-month and 98.2% at 3-month. Furthermore, we observed that Yap cKO inhibited proliferation as well as promoted abnormal differentiation and senescence of the lens epithelial cells, which ultimately led to abnormal lens structure and cataract formation. Thus, our findings revealed that Yap plays a crucial role in the development of cataract, which might be a potential target for cataract treatment.

## MATERIALS AND METHODS

### Animals

Mice were maintained under a 12-h light/dark cycle at 23 °C and were provided with food and water *ad libitum* in the Animal Care Facility at the Institute of Biophysics (Beijing, China). All experiments involving animals were approved by and conformed to the guidelines of the Institutional Animal Care and Use Committee at the Institute of Biophysics of the Chinese Academy of Sciences (Beijing, China).

### Generation of Yap conditional knockout mice

The *Yap^flox/flox^* mice were crossed with *Yap^flox/+^*; GFAP-Cre mice, and the offspring *Yap^f/f^*; GFAP-Cre (Yap cKO) mice and *Yap^f/f^* (WT) mice were used in the experiments. This approach enabled Cre recombinase to inactivate the Yap gene specifically in cells in which the GFAP promoter is active. The floxed Yap gene was identified *via* PCR using primer-1 (5′-AGTCTGTAACAACCAGTCA GGGA -3′), primer-2 (5′-GGCACTGTCAATTAATGG GC-3′) and primer-3 (5′-TCCATTTGTCCTCATCTCTT ACTAAC -3′) yielding PCR products of 550 and 600 bp for the WT and floxed alleles, respectively. For PCR of the GFAP-Cre allele, we used the forward primer (5′- GA TCTCCGGTATTGAAACTCCAGC-3′) and the reverse primer (5′-GCTAAACATGCTTCATCGTCGG-3′), yielding a 500-bp product.

### Immunohistochemical assays

Animals were euthanized by overdose of CO_2_ and their whole eyes removed. Tissues were fixed overnight (O/N) in 4% paraformaldehyde at room temperature, processed, and frozen or embedded in paraffin. Serial sections were cut at 5 μm (paraffin) or 15 μm (frozen) and either used for hematoxylin and eosin (H&E) staining or immunohistochemical analysis. Visualization and imaging were performed with a Nikon Tie-A1 confocal microscope (Nikon Instruments Inc., Melville, NY, USA) and NanoZoomer Digital Pathology software (Hamamatsu, Iwata City, Shizuoka Pref., Japan).

### β-galactosidase staining

Specimens for frozen sectioning were embedded in Tissue-Tek OCT (4583, Sakura Finetek USA Inc., Torrance, CA, USA) and quick-frozen with liquid nitrogen. Sections were cut at 15 μm and immediately mounted on Fisher Superfrost Plus slides (ZLI-9506, ZSGB-BIO, Beijing, China). Senescence-associated-β-galactosidase (SA-β-gal) was detected using the Cellular Senescence Assay Kit (C0602, Beyotime Biotechnology, Shanghai, China) following the manufacturer's protocol.

### Antibodies

Primary antibodies used were: Yap (NB110-58358, NOVUS Biologicals,Littleton, CO, USA), GFAP (MAB360, Merck & Millipore, Billerica, MA, USA), Nestin (MAB353, Merck & Millipore), AQP0 (05-321, Merck & Millipore), Ki67 (ab15580, Abcam, Cambridge, MA, USA), Caspase1 (ab108362, Abcam), β-actin (60008-1-1, Proteintech Group, Wuhan, HB, China), β-Tubulin (CW0098A, CWbiotech, Beijing, China).

Secondary antibodies used were: TRITC AffiniPure Goat Anti-Rabbit (111-025-003, Jackson Immuno-Research, Jennersville, PA, USA), Alexa Fluor 488 AffiniPure Goat Anti-Rabbit (111-545-003, Jackson ImmunoResearch), Alexa Fluor 488 AffiniPure Goat Anti-Mouse (115-545-003, Jackson Immunoresearch), Vectastain Elite ABC kit (PK-4001, ZSGB-BIO).

### Cell culture and transfection

αTN4 cells were cultured in Dulbecco’s Modified Eagle’s Medium (DMEM, Invitrogen, Waltham, MA, USA) containing 10 % (*v/v*) FBS and 50 units/mL of each penicillin and streptomycin. Cells were kept at 37 °C and 5% CO_2_. Lipofectamine 2000 reagent (Invitrogen) was applied for transfection in αTN4 cells according to the manufacturer’s instructions. The siRNA sequences used are listed as follows (5’-3’):

siCtrl: UUCUCCGAACGUGUCACGUTT
siYap1#: CUGCAGGAAGCGCUGAGUUTT
siYap2#: GUCUGCAGGAAGCGCUGAGTT

**Figure 1.**
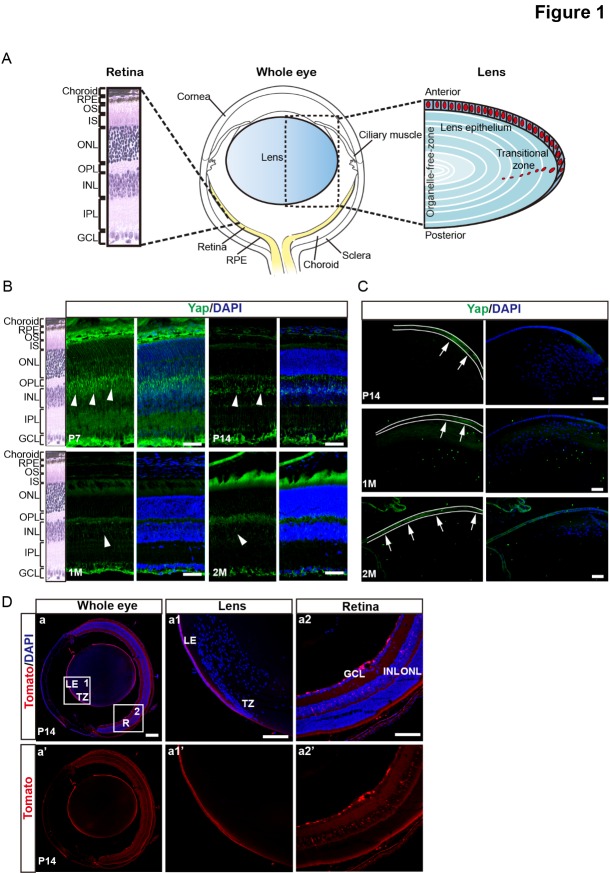
The expression patterns of Yap and GFAP-Cre recombinase in postnatal mouse eyes. (A) Schematic of a transverse section of mouse eye. (B-C) Immunostaining with anti-Yap antibody (green) on frozen eye sections at different ages. Nuclei were counterstained with DAPI (blue). Yap staining was detected in scattered cells within the INL (arrowheads) and GCL of the retina and the lens epithelium (arrows). (D) Cre recombinase (red) was expressed in the lens epithelium and INL, GCL of retina in frozen eye sections of *Tomato^f/+^*; GFAP-Cre mice at P14. Nuclei were counterstained with DAPI (blue). LE, lens epithelium; TZ, transitional zone; RPE, retinal pigment epithelium; OS, outer segment; IS, inner segment; ONL, outer nuclear layer; OPL, outer plexiform layer; INL, inner nuclear layer; IPL, inner plexiform layer; GCL, ganglion cell layer. Scale bars: 25 μm (B-C), 100 μm (D).

### ATP detection

ATP levels were determined using a bioluminescent ATP assay kit (Promega, Madison, WI, USA) according to the manufacturer's instructions.

### RNA extraction, reverse transcription and quantitative real time PCR (qRT-PCR)

Total RNA was isolated from cells using TRIzol reagent (Invitrogen), and reverse transcription was performed using the one step first strand cDNA synthesis kit (AT311, TransGen Biotech, Beijing, China). qRT-PCR was performed using 2x SYBR Green PCR master mix (CW2601, CWbiotech) in an Agilent Mx3005P qRT-PCR system. The melting temperature profiles of the final products were used to ensure amplicon specificity. The relative fold-change in the expression of each mRNA was calculated using the ddCt method relative to the expression of Actin. The mouse qRT-PCR primers used are listed as follows:

Yap-5F: TACTGATGCAGGTACTGCGG
Yap-3R: TCAGGGATCTCAAAGGAGGAC
Taz-5F: CATGGCGGAAAAAGATCCTCC
Taz-3R: GTCGGTCACGTCATAGGACTG
Crygc-5F: AATGCGGCTGTATGAGAAAGAA
Crygc-3R: GGAAGCGCCGGTACTCTTG
Crygd-5F: CGGCTCTCACAGGATCAGACT
Crygd-3R: GGTAGTTGGTCATGTCGTAGAGG
Sox2-5F: GCGGAGTGGAAACTTTTGTCC
Sox2-3R: CGGGAAGCGTGTACTTATCCTT
Pax6-5F: TACCAGTGTCTACCAGCCAAT
Pax6-3R: TGCACGAGTATGAGGAGGTCT
Dnase2b-5F: ACACCAGAAATCTCATGCAGAAA
Dnase2b-3R: GGAGTCCAGGTACAGGTACTG
Il6-5F: TCTATACCACTTCACAAGTCGGA
Il6-3R: GAATTGCCATTGCACAACTCTTT
Tnfα-5F: CAGGCGGTGCCTATGTCTC
Tnfα-3R: CGATCACCCCGAAGTTCAGTAG
Cdk6-5F: TCTCACAGAGTAGTGCATCGT
Cdk6-3R: CGAGGTAAGGGCCATCTGAAAA
p21-5F: GTGGGTCTGACTCCAGCCC
p21-3R: CCTTCTCGTGAGACGCTTAC
p53-5F: GCGTAAACGCTTCGAGATGTT
p53-3R: TTTTTATGGCGGGAAGTAGACTG

### RNA-Seq

Lenses from WT and Yap cKO mice were dissected. RNA was extracted for RNA-Seq according to standard protocols. EdgeR [18, 19] was used to evaluate the statistical significance of differentially transcribed genes. The Gene Ontology (GO) enrichment analysis was performed using the Enrichr (http://amp.pharmmssm.edu/Enrichr/) [20, 21].

### Statistical analysis

The data were presented as the mean ± SEM. Statistical analyses were performed using the GraphPad Prism 7 software (San Diego, CA, USA). Student’s t-test or Two-way RM ANOVA was used to determine significance (*P < 0.05, **P < 0.01).

**Figure 2.**
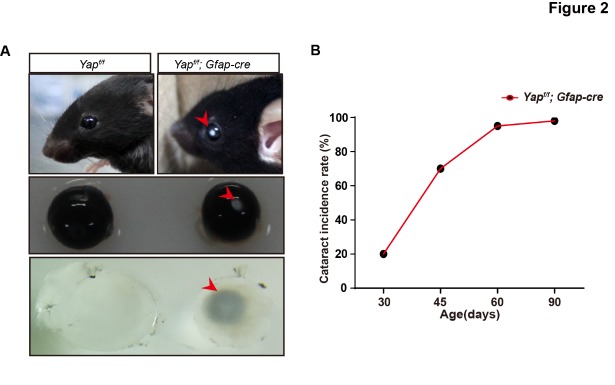
Cataract occurs in 1.5-month-old Yap-deficient mice. (A) 1.5-month-old *Yap^f/f^*; GFAP-Cre mouse demonstrating severe nuclear cataract, with opaque cloudiness (arrowheads) in lens. (B) Incidence and latency of cataract formation in *Yap^f/f^*; GFAP-Cre mice.

**Figure 3.**
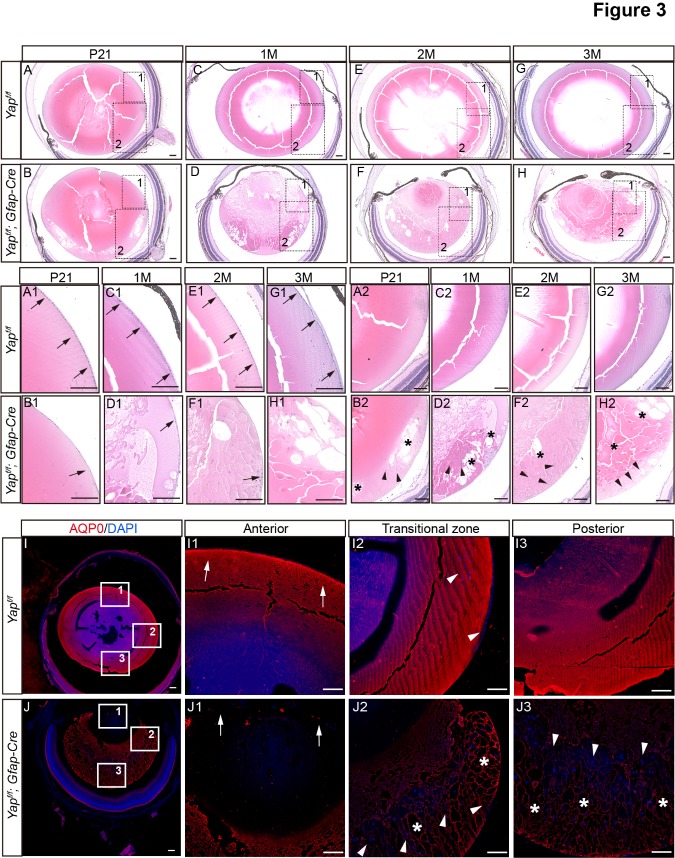
Abnormal lens structure in Yap-deficient mice at different stages. (A-H) H&E staining of lens from *Yap^f/f^* and *Yap^f/f^*; GFAP-Cre mice at P21, 1-month, 2-month and 3-month, respectively. (A1-H1) Selected high magnification views of lens epithelium (A-H), showed the disorganization of anterior lens epithelium (arrows indicate lens epithelial cells) of *Yap^f/f^*; GFAP-Cre lens. (A2-H2) High magnification views of transitional zone regions (A-H), showed the accumulation of ectopic cells and morgagnian globules at the transitional zone and posterior region of *Yap^f/f^*; GFAP-Cre lens (asterisks indicate morgagnian globules). Arrowheads indicate hematoxylin-positive cells. (I-J) Immunofluorescent staining revealed the abnormal distribution of AQP0 in Yap-deficient lens compared with wildtype (1.5-month). Asterisks indicate morgagnian globules and arrowheads indicate displaced cell nuclei. Scale bars: 100 μm.

### RESULTS

#### Expression of Yap and GFAP Cre recombinase in postnatal mouse eyes

To investigate the role of Yap in mouse eyes, we first defined the expression of Yap in eyes by immunofluorescent staining and found that Yap expression was restricted to the lens epithelium (Fig. 1C), inner nuclear layer (INL), and ganglion cell layer (GCL) of the retina (Fig. 1B). It has been reported that Cre recombinase under the control of the GFAP gene promoter (GFAP-Cre) is mainly expressed in the developing lens epithelium starting at E18 [22], when the formation of the lens vesicle is almost complete [23]. To analyze the expression pattern of GFAP-Cre, we utilized R26R Tomato reporter mice for lineage-tracing of GFAP positive cells and found that GFAP Cre recombinase was mainly expressed in lens epithelium, INL and GCL of the retina, which was similar to Yap’s expression pattern (Fig. 1D and Fig. S1). Taken together, the Yap conditional knockout mice (Yap cKO, *Yap*^f/f^; GFAP-Cre) provide us a tool to investigate the role of Yap in mouse eyes, especially in the lens epithelium.

**Figure 4.**
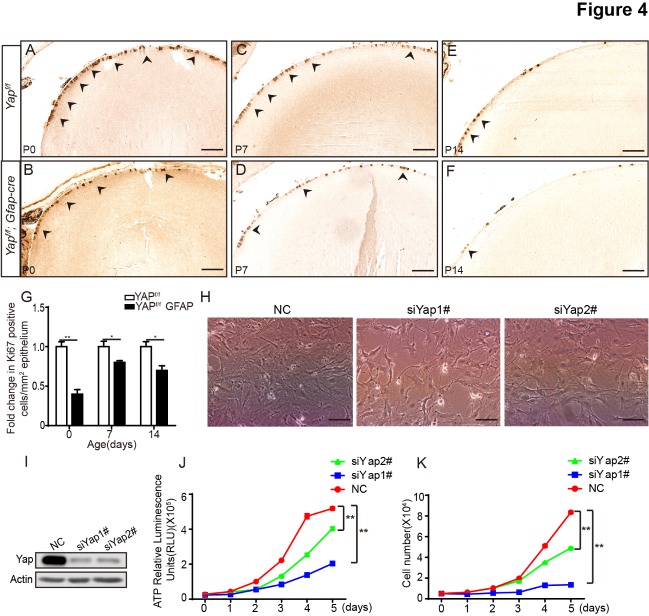
Yap deficiency suppresses cell proliferation *in vivo* and *in vitro.* (A-F) The Ki67 positive ratio of lens epithelial cells decreased in Yap-deficient mice at different stages (arrowheads indicate Ki67 positive cells). (G) The relative number of Ki67 positive lens epithelial cells (number of Ki67 positive lens epithelial cells / lens epithelium area). The data are shown as mean ± S.E.M. (Student’s *t*-test, *P<0.05, **P<0.01, n=10). (H-I) Knockdown efficiency of Yap in αTN4 cell using siRNA. (J-K) Cell viability and growth assay revealed that proliferation was downregulated in Yap knockdown αTN4 cells. The data are shown as mean ± S.E.M. (Two-way RM ANOVA, **P<0.01, n=5). Scale bars: 50 μm.

**Figure 5.**
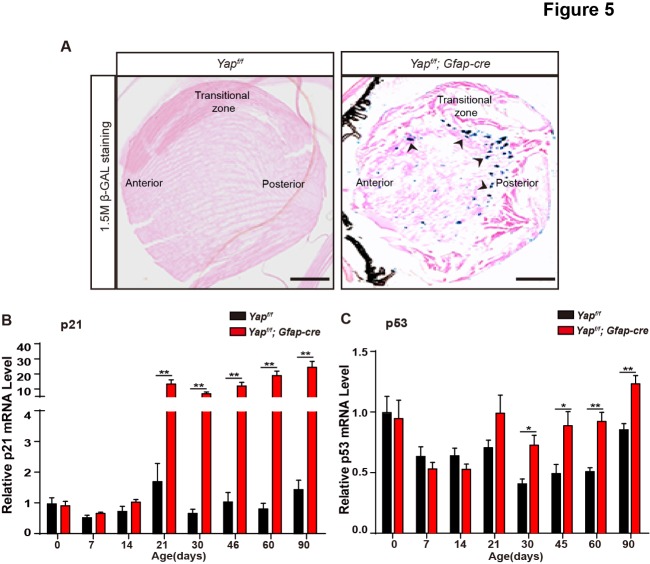
Yap deficiency results in cellular senescence in the lens. (A) Frozen eye sections from 1.5-month-old *Yap^f/f^* and *Yap^f/f^*; GFAP-Cre littermate mice were stained for SA β-galactosidase activity (arrowheads). (B and C) qRT-PCR analysis of p21 and p53 relative mRNA levels in the lens from *Yap^f/f^* and *Yap^f/f^*; GFAP-Cre littermate mice at different stages. The data are shown as mean ± S.E.M. (Student’s *t*-test, *P<0.05, **P<0.01, n=6). Scale bars: 50 μm.

#### Yap deficiency in GFAP positive cells leads to cataract

In GFAP-Cre mediated Yap cKO, the expression of Yap was greatly decreased in the lens but not the retina (Fig. S2A-C). The mRNA level of Yap in the lens was significantly reduced at P7 (Fig. S2D). Interestingly, Yap deficient mice developed lens opacities at 1.5-month of age (Fig. 2A), the lenses were smaller than that of wild-type (WT, *Yap*^f/f^) and manifested a typical nuclear cataract phenotype - central opacity surrounded by a relatively transparent cortical region (Fig. 2A). This cataract became progressively severe as the mouse aged (Fig. 2B).

Histological changes in the eyes were visualized by H&E staining from WT or Yap cKO mice at different stages. We found that the morphology of Yap deficient eyes appeared normal before 21-days of age (Fig. S3). However, Yap deficiency led to a dramatic age-dependent cataract development (Fig. 3A-H), specifically, 1) a decreased number of lens epithelial cells from 21-day to 3-month (Fig. 3A1-H1); 2) an accumulation of morgagnian globules from 21-day to 3-month (Fig. 3A2-H2); 3) a disordered denucleation featured with increased numbers of hematoxylin positive cell in the transitional zone and the posterior area of the lens from 21-day to 3-month (Fig. 3A2-H2); 4) a cloudy organelle-free-zone and decreased size of the lens from 1-month to 3-month (Fig. 3C-H). There was no significant abnormality in the retina of Yap deficient mice (Fig. S4). Next, we used aquaporin-0 (AQP0) immunofluorescence staining to examine the lens structure. We found that AQP0 was exclusively and evenly localized in fiber cell membranes in WT lens (Fig. 3I). In contrast, within Yap cKO lens (Fig. 3J), AQP0 was not detected in the organelle-free-zone (Fig. 3J1) and was markedly reduced in the transitional zone and the posterior lens (Fig. 3J2-J3). Similar to H&E staining, we observed an abnormal denucleation in the transitional zone and the posterior lens (Fig. 3J2-J3). Taken together, morphological and histological analysis of the lens from Yap cKO mice clearly demonstrated that Yap deficiency leads to cataract features, including reduced number of lens epithelial cells, abnormal fiber cell denucleation and morgagnian globules accumulation.

#### Yap is essential for the proliferation of lens epithelial cells

Lens epithelial cells maintain the ability to proliferate throughout a mammal’s lifespan, which is essential for the lens function. Previous studies showed that the Hippo signaling pathway plays an important role in cell proliferation [24-27]. Here, we found that there is a significant decrease in the number of lens epithelial cells in Yap cKO mice (Fig. 3A1-H1). We performed Ki67 immunohistochemical staining to further determine the role of Yap in lens epithelial cells proliferation. We found that the number of Ki67 positive lens epithelial cells was significantly decreased in Yap cKO lenses at 0, 7, 14-postnatal day (Fig. 4A-G). Moreover, we used αTN4 cells to examine the role of Yap on cell proliferation in vitro. Yap knockdown in αTN4 cells strongly suppressed cell proliferation (Fig. 4H-K). Taken together, our results reveal that Yap deficiency inhibits the proliferation of lens epithelial cells *in vivo* and *in vitro.*

**Figure 6.**
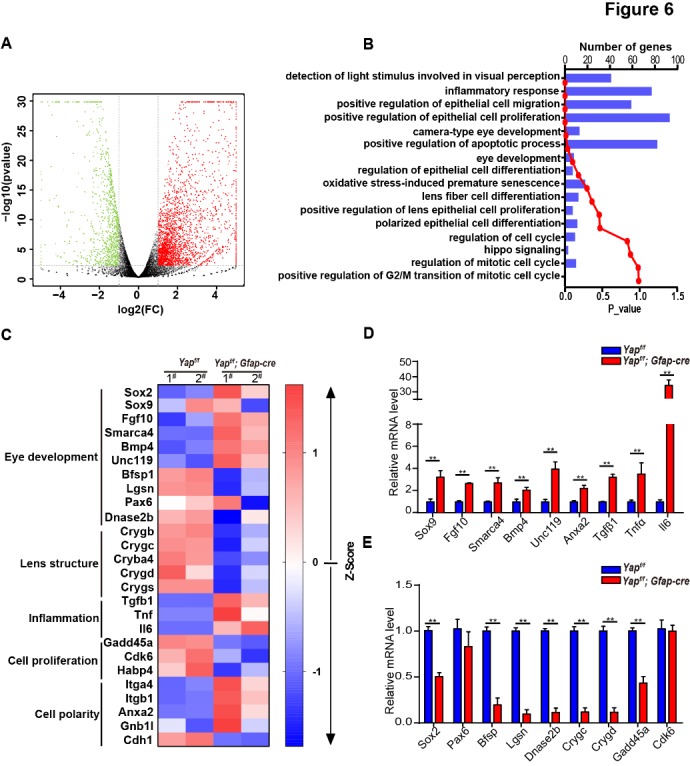
Analysis of differential gene expression in Yap-deficient mice. (A) Volcano map of significantly expressed genes in the lens from 1.5-month-old *Yap^f/f^* and *Yap^f/f^*; GFAP-Cre littermate mice. Red dots and green dots represent significantly up-regulated and down-regulated genes, respectively (*P*<0.01 and fold change >2). (B) The distributions of biological processes (BP). The curve indicates p-value of BP terms and the bar graph indicates the gene number distribution in BP terms. (C) The list of 24 significant transcripts that are regulated by Yap deficiency were classified into five categories including eye development, lens structure, inflammation and cell proliferation as well as polarity determination. (D-E) Secondary validation of the differentially expressed genes with qRT-PCR analysis. The data are expressed as mean ± S.E.M. (Student’s *t*-test, **P<0.01, n=6).

**Figure 7.**
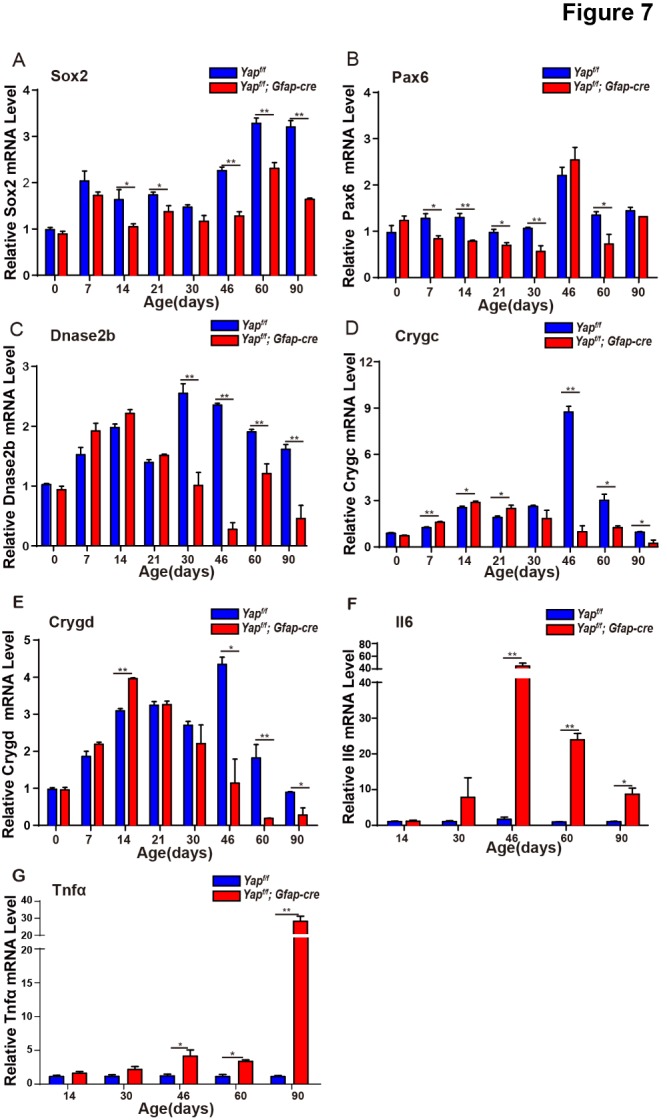
Secondary validation of differentially expressed genes in Yap-deficient lenses at different stages. (A-G) qRT-PCR analysis of relative Sox2, Pax6, Dnase2b, Crygc, Crygd, Il6 and Tnfα mRNA levels in lens from *Yap^f/f^* and *Yap^f/f^*; GFAP-Cre littermate mice at different stages. The data are shown as mean ± S.E.M. (Student’s *t*-test, **P<0.01, n=6).

#### Yap deficiency in GFAP positive cells leads to senescence in lens

Early cataract is accompanied by accumulated morgagnian globules and cellular senescence [28]. Furthermore, inhibition of cellular senescence could postpone cataract formation [29]. In our previous study, we found that Yap is crucial for the regulation of cellular senescence in normal diploid cells [30]. Here, we observed that morgagnian globules accumulation, a process associated with lens senescence, occurred in Yap cKO lens (Fig. 3B). Thus, we examined the senescence-associated β-galactosidase activity in the lens and found that β-galactosidase activity was significantly increased in 1.5-month-old Yap cKO compared to WT lens (Fig. 5A). Moreover, we examined the expression levels of p21, p53 and p16 in the lens using quantitative RT-PCR (qRT-PCR) and found that mRNA levels of p21 and p53 were significantly increased in Yap cKO mice (Fig. 5B-C). Taken together, these results suggest that Yap deficiency in GFAP positive cells increased the mRNA levels of senescence-associated genes and promoted cellular senescence in lens.

#### RNA profiles in Yap cKO lens

To further illustrate the molecular mechanism underlying cataract formation induced by Yap deletion, we examined RNA expression profiling using RNA-seq in the lenses from WT and Yap cKO mice at 1.5-month-old. A set of differentially expressed genes have been identified in Yap deficient mice *versus* age-matched littermate controls (Fig. 6A). Gene Ontology (GO) enrichment analysis showed that these differentially expressed genes were frequently enriched in biological processes such as epithelial cell proliferation, differentiation and migration, inflammatory response, camera-type eye development as well as apoptotic process (Fig. 6B). These altered genes were also functionally linked with eye development, lens structure, inflammation, cell proliferation and polarity (Fig. 6C). Furthermore, the expression of 24 genes were validated using RT-qPCR (Fig. 6D-E) and we found that: 1) the expression levels of Sox2 and Pax6, which are important for lens development and cataract formation [31, 32], were significantly decreased in Yap cKO lens (Fig. 7A-B); 2) Dnase2b, an enzyme of DNA degradation was significantly downregulated upon Yap knockout from 21-days of age, which correlates with abnormal denucleation of fiber cells (Fig. 7C); 3) Crystallins, the lens structure proteins, were dramatically reduced in Yap cKO lens (Fig. 7D-E); 4) Inflammation genes such as Tnfα and Il6 were significantly increased in Yap cKO lens (Fig. 7F-G). Taken together, Yap deficiency led to the reduced expression levels of Sox2, Pax6 and Dnase2b at an early stage, which may initiate cataract; and at a later stage, the downregulation of lens structural genes and increased inflammation might exacerbate the cataract process. Therefore, we argue that Yap regulates the expression of genes related to lens development and Yap deficiency leads to abnormal lens development and cataract formation.

**Figure 8.**
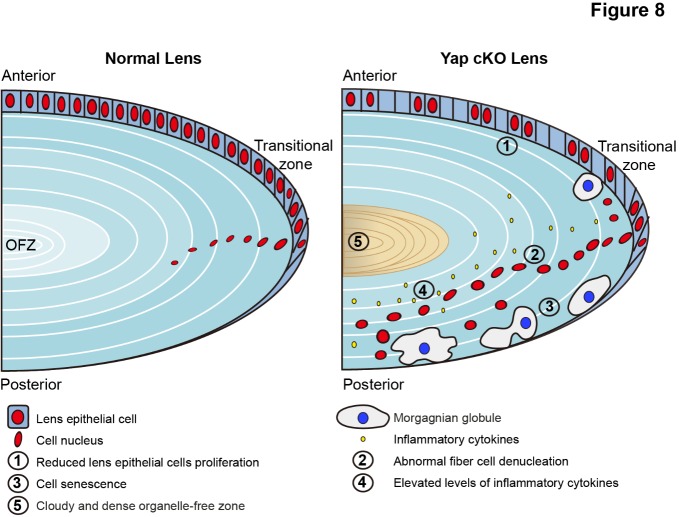
Proposed model of cataract formation in Yap conditional knockout mice. The model proposes that Yap deficiency in the lens led to a cascade of events, including: 1) reduced cell proliferation; 2) abnormal denucleation of fiber cells; 3) cellular senescence; 4) increased inflammation; 5) and consequently, cataract with cloudy and dense organelle-free-zone.

### DISCUSSION

Cataract is the most common disease that causes blindness and its complicated etiopathogenesis limits effective therapies. Therefore, it is of great medical significance to explore the mechanisms of cataract formation and progression so that a novel therapeutic target(s) can be developed to prevent or treat cataract. Here, we analyzed the temporal and spatial expression patterns of Yap in the murine lens. We found that Yap is mainly expressed in the lens epithelium. GFAP-Cre mediated Yap knockout in the lens leads to abnormal lens structure including morgagnian globules accumulation, reduced lens epithelial cells and abnormal denucleation in the transitional zone and posterior of the lens. Further mechanistical studies indicated that (1) Yap knockout inhibited the proliferation of lens epithelial cells *in vivo* and *in vitro*; (2) Yap deficiency led to senescence in lens; (3) Sequential denucleation of fiber cells was disordered in Yap deficient mice; (4) Increased inflammation in the lens upon Yap knockout (Fig. 8). Additional transcriptional profiling analysis found that the downregulated Sox2 and Pax6 at early stages might initiate cataract, and the increased inflammation factors and decreased levels of lens structural proteins in the later stages could exacerbate cataract formation. Collectively, our data reveals a novel function of Yap in the lens and links Yap deficiency with the development of cataract, with an implication of a diagnostic and therapeutic target for cataract.

This study is the first to demonstrate that Yap deficiency causes cataract in mice. Here, we categorized the pathological alterations of Yap deficiency-induced cataract into two stages - the primary effects and the secondary effects. The primary effects include the decrease in proliferation of lens epithelial cells and in this stage, Yap deficiency downregulated the expressions of genes related to lens proliferation and development-related genes (Sox2, Pax6 and Dnase2b), which initiated cataract. In the secondary stage, Yap deletion accelerated cellular senescence and reduced the expression levels of lens structural proteins as well as increased inflammation, which finally promoted cataract formation.

The initial disorder of cataract development is the suppressed proliferation of lens epithelial cells in Yap cKO mice, which leads to fewer lens epithelial cells from P21. Lens epithelial cell has been reported to be essential for lens development and its decreased number leads to cataract in alpha-crystalline knockout or FoxE3 knockout mice [33, 34]. Further RNA-seq and subsequent RT-qPCR analysis reveal the downregulation of Sox2 and Pax6 in the early stage, which could be the molecular mechanism underlying the decreased proliferation. Moreover, as key regulators in lens development, Sox2 and Pax6 could activate a battery of genes for early lens development such as δ1-crystallin [35]. Mutations of Sox2 in mice leads to anophthalmia or microphthalmia [31] and patients with Sox2 mutation are more likely to develop posterior cortical cataract [32]. In addition, Yap could also transcriptionally induce Sox2 expression through physically interacting with transcription factor Oct4 in stem-like cells from non-small cell lung cancer [36]. We also identified Sox2 as a key molecule in Yap deficient lens by functional analysis with the MetaCore software. Therefore, in addition to decreased proliferation, Yap-deficiency downregulated Sox2/Pax6 expression that might lead to developmental dysfunction in the lens.

During lens development, optical clarity is ensured by the degradation of intracellular nuclei in fiber cells, in which Dnase2b plays an important role [37]. Many reports showed that the decrease of Dnase2b expression is closely related to cataract formation [38-40]. In our study, the mRNA level of Dnase2b in Yap deficient lens decreased at P21, indicating Yap could transcriptionally regulate Dnase2b during lens development and Yap deficiency-induced downregulation of Dnase2b led to the abnormal denucleation and finally cataract.

We have reported that Yap transcriptionally regulates Cdk6 in diploid cells and knockdown of Yap induces cellular senescence [30]. However, we observed that Cdk6 level was decreased only at P7 (Fig. S5), suggesting that Cdk6 might not be a main target in lens development. We further found that β-galactosidase activity was significantly increased in Yap deficient lens, and the mRNA levels of p21 and p53 were also significantly elevated since P21, which is correlated with senescence-induced morgagnian globules accumulation during cataract formation.

Many studies show that inflammation [9, 41, 42] and mutation of crystalline proteins [43-45] are involved in the etiology of cataract. We also observed that there were increased levels of inflammation factors and the decreased mRNA levels of lens structural genes in Yap deficient lens at P30. Thus, we argue that inflammation further accelerates cataract formation. However, whether Yap deficiency directly or indirectly induced inflammation remains to be further investigated.

Yap and Taz are twin homologues in the mammalian system and mostly have redundant functions. In our study, we found that the lenses in GFAP-mediated Taz cKO mice are normal. Similar to Yap cKO alone, GFAP-mediated Taz and Yap double cKO mice showed cataract phenotype (Fig. S6). Previous studies have demonstrated that Yap and Taz also have distinct roles. For example, Yap global knockout is embryonically lethal. However, Taz conventional knockout mice only exhibit some developmental defects such as polycystic kidney and emphysema [46-48]. In addition, Yap and Taz exert different biological functions in myogenic differentiation and they are differently regulated through distinct interaction with Parafibromin [49, 50]. Not surprisingly, we indeed found that the expression level of Taz in mouse lens was much lower than Yap. Our results reveal that Yap, rather than Taz, plays a critical role in lens development and cataract formation.

As GFAP-Cre recombinase is also expressed in the INL and GCL of retina, it is possible that the cataract that occurred in the Yap cKO model may secondary to Yap cKO-induced retinal defects. However, the general retinal morphology was not altered in Yap cKO mice. Additionally, we observed that the gross brain structure in Yap cKO mice appears normal. Therefore, we argue that Yap cKO lens phenotypes are autonomously developed (Fig. S4).

In summary, our results show that Yap plays an essential role in the formation of cataract in the mouse. The pathological examination and transcriptional profiling analysis provide insight into the development of cataract as a consequence of Yap deficiency.

## Supplementary Materials

The Supplemenantry data can be found online at: www.aginganddisease.org/EN/10.14336/AD.2018.0910
