# Supplementary figures and images for: Deficiency of Yes-Associated Protein Induces Cataract in Mice
Source: Aging Dis. 2019 Apr 1;10(2):293–306. doi: 10.14336/AD.2018.0910 (PMC6457047; doi:10.14336/AD.2018.0910)

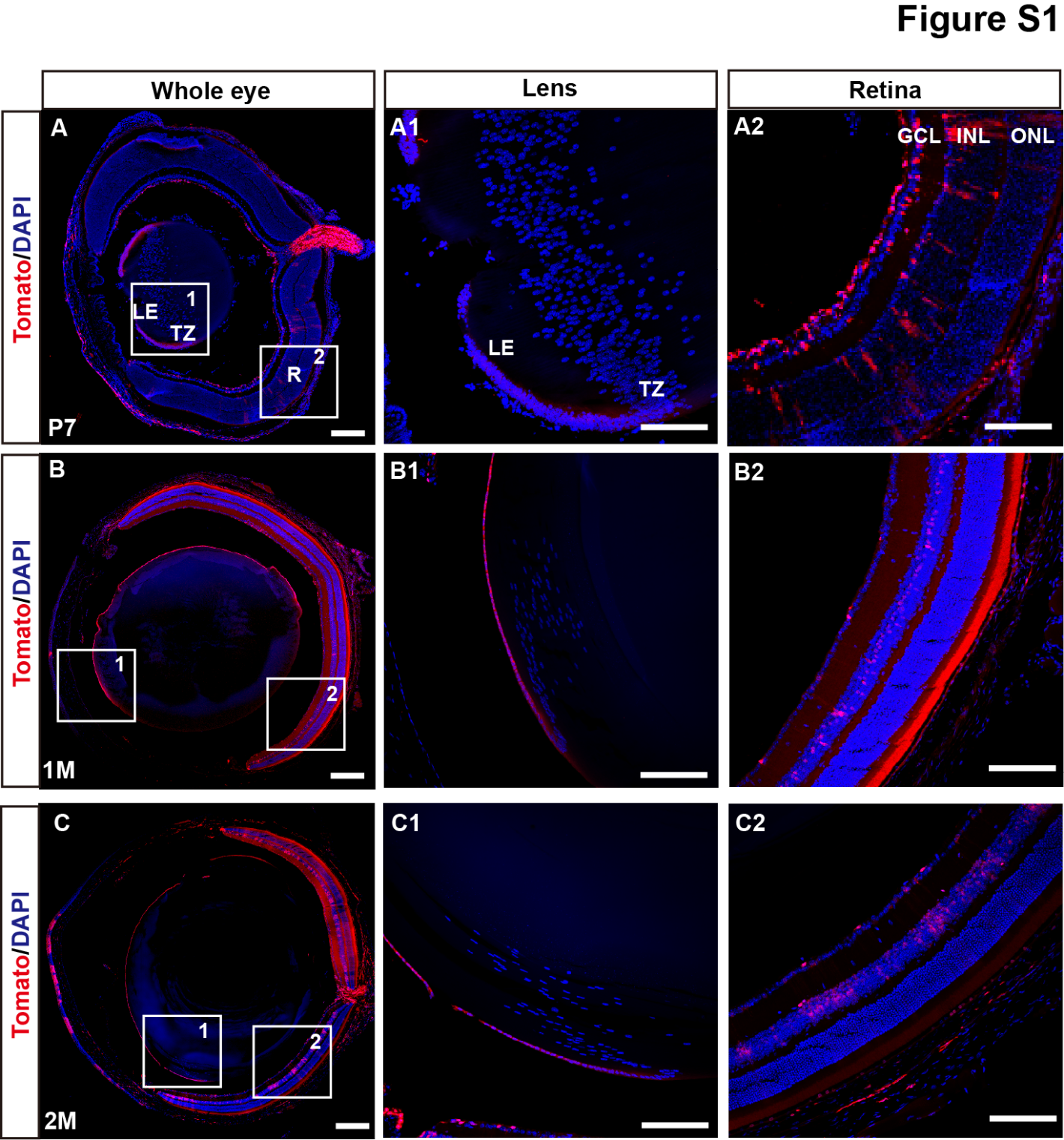

Supplement: Supplementary file 1 — The Supplemenantry data can be found online at: www.aginganddisease.org/EN/10.14336/AD.2018.0910 [file ad-10-2-293-s-g1.tif]
